# FEV_1_ inversely correlates with metalloproteinases 1, 7, 9 and CRP in COPD by biomass smoke exposure

**DOI:** 10.1186/1465-9921-15-74

**Published:** 2014-06-30

**Authors:** Martha Montaño, Raul H Sansores, Carina Becerril, Jose Cisneros, Georgina González-Avila, Bettina Sommer, Leticia Ochoa, Iliana Herrera, Alejandra Ramírez-Venegas, Carlos Ramos

**Affiliations:** 1 Departamento de Fibrosis Pulmonar, Calzada de Tlalpan 4502, Tlalpan D.F. México, C.P. 14080 México, DF, Mexico; 2 Departamento de investigación en Tabaquismo, Calzada de Tlalpan 4502, Tlalpan D.F. México, C.P. 14080 México, DF, Mexico; 3 Departamento de Enfermedades Crónico Degenerativas, Calzada de Tlalpan 4502, Tlalpan D.F. México, C.P. 14080 México, DF, Mexico; 4 Departamento de Hiperreactividad Bronquial, Instituto Nacional de Enfermedades Respiratorias Ismael Cosío Villegas, Calzada de Tlalpan 4502, Tlalpan D.F. México, C.P. 14080 México, DF, Mexico

**Keywords:** Biomass combustion exposure, C-reactive protein, Chronic obstructive pulmonary disease, FEV_1_, Metalloproteinases, Tobacco smoking

## Abstract

**Background:**

Matrix metalloproteinases (MMPs) and C-reactive protein (CRP) are involved in chronic obstructive pulmonary disease (COPD) pathogenesis. The aim of the present work was to determine plasma concentrations of MMPs and CRP in COPD associated to biomass combustion exposure (BE) and tobacco smoking (TS).

**Methods:**

Pulmonary function tests, plasma levels of MMP-1, MMP-7, MMP-9, MMP-9/TIMP-1 and CRP were measured in COPD associated to BE (n = 40) and TS (n =40) patients, and healthy non-smoking (NS) healthy women (controls, n = 40).

**Results:**

Plasma levels of MMP-1, MMP-7, MMP-9, and MMP-9/TIMP-1 and CRP were higher in BE and TS than in the NS healthy women (*p <0.01*). An inverse correlation between MMP-1, MMP-7, MMP-9, MMP-9/TIMP-1 and CRP plasma concentrations and FEV_1_ was observed.

**Conclusions:**

Increase of MMPs and CRP plasma concentrations in BE suggests a systemic inflammatory phenomenon similar to that observed in COPD associated to tobacco smoking, which may also play a role in COPD pathogenesis.

## Introduction

Chronic obstructive pulmonary disease (COPD) is a leading cause of mortality and morbidity worldwide [1]. Although tobacco smoking is well recognized as the major risk factor for the disease, exposure to biomass smoke (BE) and other fuel combustion products has also been described as an additional risk factor [2,3]. The association between BE, mainly wood smoke and COPD in different populations, particularly in developing countries where wood is used as fuel for cooking and heating, has been established [4-6]. The clinical profile of COPD associated with BE and its prognostic factors have been described in Mexican population [3,5]. Women are more susceptible than men to this disease exhibiting bronchial symptoms, decreased exercise capacity, changes in quality of life, and augmented use of healthcare services and supplemental oxygen. Nevertheless, a lot of questions about its pathogenesis and molecular mechanisms associated with biomass combustion exposure remain unanswered [2,3].

Several recent reports have suggested that a persistent, low-level, systemic inflammation plays a significant pathogenic role in COPD. Accordingly, elevated circulating levels of C-reactive protein (CRP) among other inflammatory markers [7], such as plasma and sputum matrix metalloproteinases (MMPs) and the tissue inhibitor of metalloproteinase-(TIMP)-1 levels have been reported, suggesting their participation in the pathogenesis of COPD secondary to TS [8-11].

Despite the growing evidences of BE as a risk factor for COPD, very little information on systemic inflammation and pathogenic mechanisms has been described [6,7] and so far, there are few studies describing inflammatory molecules associated to changes in FEV_1_ in COPD due to BE.

The aim of the present study was to determine plasma concentrations of matrix metalloproteinase-(MMP)-1, MMP-7, MMP-9, MMP-9/TIMP-1, and CRP in COPD associated with BE, specifically to wood smoke. A group of non-smoking (NS) healthy women was considered as control group and data were compared with subjects having COPD associated to tobacco smoke (TS).

## Materials and methods

### Study population

Eighty women with a clinical and functional diagnosis of COPD associated with BE or tobacco smoke were recruited from the COPD Clinic from January to December 2013. The quantity of tobacco smoked and the degree of exposure to BE was determined by a clinical interview, using the Spanish version of a validated instrument that was modified to include additional questions directly related to fuels used for cooking and heating [12]. The main inclusion criterion was a history of daily wood smoke exposure for at least 200 hours/year or a history of tobacco smoking of at least >10 pack/year. Cumulative exposure to wood smoke was expressed as hours/year, which was calculated by multiplying the number of years of cooking with wood by the average of daily hours spent cooking [5].

COPD diagnosis was confirmed by medical history and spirometry results, which were interpreted according to the GOLD criteria [13]. We excluded from the study groups those subjects who had both BE and tobacco exposure or a history of other chronic pulmonary conditions such as asthma, tuberculosis or bronchiectasis. Patients with COPD that participated in this study were clinically stable, with no history of exacerbations for at least 6 weeks prior to the study. Forty healthy non-smoking (NS) women volunteers with normal spirometry values, without a history of tobacco smoking or biomass smoke exposure, with no signs of infectious respiratory disease during the past 3 weeks and no history of asthma, allergy or other diseases were considered as the control group.

Informed consent was obtained from each subject and the protocol was approved by the local Ethic and Research Committees at the Instituto Nacional de Enfermedades Respiratorias Ismael Cosío Villegas (Protocol number B 25-12).

### Measurements

#### Pulmonary function tests

All subjects were evaluated through spirometry both pre- and post-bronchodilator following the procedures recommended by the American Thoracic Society/European Respiratory Society [14]; accordingly, a dry rolling-seal volume spirometer (Sensormedics, Yorbalinda, CA, USA) was used, and Mexican standard reference equations were applied [15]. These reference equations are similar to the National Health and Nutrition Examination Survey III values for Mexican-Americans [16].

Diagnosis of COPD was established according to the history of tobacco smoking or wood smoke exposure and pulmonary function tests after inhalation of 400 μg of salbutamol [13].

Venous blood samples were collected from COPD patients and NS in 5 mL lithium heparin-coated tubes, centrifuged and plasma protein content was measured by the bincinchoninic acid protein assay (Pierce Chemical Company, Rockford, IL, USA) [17]. Plasma samples were stored at -70°C until analyzed.

### Plasma MMPs and CRP quantification

Concentrations of plasma MMP-1, MMP-7, MMP-9, MMP-9/TIMP-1 complex (duo set) and CRP were determined by commercially available ELISA kits (R&D Systems, Minneapolis, MN, USA), according to the manufacturers’ instructions. The detection limits were: 0.023 ng/mL for MMP-1, 0.016 ng/mL for MMP-7, 0.36 ng/mL for MMP-9, 10.0 pg/mL for TIMP-1 and 0.02 μg/mL for C-reactive protein.

### Statistical analysis

Data were expressed as mean ± SD for at least three independent experiments. One-way analysis of variance (ANOVA) followed by Tukey’s test were used to adjust multiple comparisons between groups. Associations between variables were performed using Pearson’s correlation coefficient (r). The SPSS software for Windows (Chicago, IL*)* was used for statistical analyses*; p <0.05* was considered statistically significant.

## Results

### Patients’ clinical characteristics

General clinical characteristics of COPD patients with BE or tobacco smoke and control subjects are shown in Table 1. Patients with BE were significantly older, shorter and with higher BMI than tobacco smoke subjects and healthy control women. The mean exposure to biomass was 230 ± 132 hours/year, whereas smokers had a mean cumulative tobacco consumption of 50 ± 30 pack/year. Despite women with BE showing a significantly lower SaO_2_, no differences were observed in the domains of the CRQ nor in the GOLD stages (Table 1).

**Table 1:** Clinical characteristics of COPD and NS healthy women (control)

	**Control n = 40**	**BE n = 40**	**TS n = 40**	***p***
**Characteristics**				
Age (years)	65 ± 10	72 ± 8^§^	69 ± 9	0.01 vs Ctrl
Height (cm)	158 ± 6	147 ± 7^§^	159 ± 10	0.01 vs TS
				0.02 vs TS
Weight (Kg)	65.9 ± 9	62 ± 11	69 ± 9^§^	0.05 vs Ctrl
BMI (Kg/m^2^)	27 ± 8	29 ± 5	26 ± 3^§^	0.01 vs BE
**Exposure and physiological characteristics**				
Tobacco index (Pack/yr)	-	-	50 ± 30	
Biomass index (Hrs/year of exposure)	-	230 ± 132	-	
FEV1 (% predicted)	107.8 ± 8.7	62.1 ± 25.6^§^	49.5 ± 23.9^§^	0.01 vs Ctrl
				0.03 BE vs TS
FVC (% predicted)	109 ± 9	78 ± 19^§^	82 ± 14^§^	0.01 vs Ctrl
FEV1/FVC ratio	91 ± 9.5	53 ± 16^§^	56 ± 15^§^	0.01 vs Ctrl
Bronchodilator response (FEV1; %)	-	9.54 ± 17	9.14 ± 8	0.883**
PaO_2_ (mmHg)	-	53 ± 7	59 ± 10	0.056**
PaCO_2_ (mmHg)	-	34 ± 4	32 ± 5	0.136**
6MWT (m)	-	274 ± 155	327 ± 142	0.075**
SaO_2_ (%)	-	88 ± 5	91 ± 5	0.039**
**Chronic respiratory questionnaire**				
Dyspnea	-	21 ± 10	17 ± 8	0.098**
Fatigue	-	19 ± 5	20 ± 5	0.711**
Master	-	37 ± 7	36 ± 9	0.570***
Control	-	22 ± 4	22 ± 5	0.988**
Total score	-	99 ± 15	95 ± 17	0.283**
GOLD stage				
I	-	8 (20)	8 (20)	0.075*
II	-	25 (63)	15 (38)	
III	-	6 (15)	12 (30)	
IV	-	1 (2.)	5 (12)	
Exacerbation rate/yr (min-max)	-	1.27 (1-3)	1.38 (1-3)	0.495**

Data are presented as mean ± SD unless otherwise indicated. Ctrl = Control; BE = biomass exposure; TS: tobacco smoking; BMI: biomass mass index; FEV_1_: forced expiratory volume in one second; FVC: forced vital capacity;% predicted: percentage of the predicted value. For GOLD stage figures are presented as number (%).

Statistical tests: ^§^ANOVA-Tukey test; *X^2^ test; **Student-T test; ***Welch test. All comparisons with Ctrl and TS were against BE after Bonferroni adjustments.

### Pulmonary function tests

Both FEV_1_ (% predicted) and FVC (% predicted) were significantly lower in women with COPD associated to smoking in comparison to control healthy women and with BE (Table 1); similarly FVC values and FEV_1_/FVC ratio were lower in TS and BE than in controls (Table 1). Finally, it is important to note that in terms of the GOLD classification, BE patients differ of TS patients because the majority (83%) of them are included within stages I and II, whereas TS patients are more homogenously distributed, although this difference was not statistically significant (Table 1, p = 0.075). No differences in the rate of exacerbations were found.

### MMPs plasma concentrations

**Figure 1:**
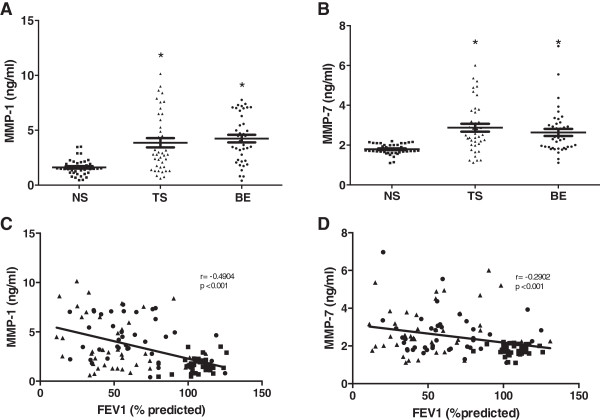
**Plasma levels of MMP-1 and MMP-7 in COPD groups compared with NS healthy women (controls), and relationship between plasma MMP-1 and MMP-7 and FEV**_**1 **_**(% predicted).** There was a significant difference between BE and TS with NS healthy subjects. No difference was found among COPD groups: **(A)** MMP-1. **(B)** MMP-1 between MMP-1 or MMP-7 and FEV_1_ (% predicted) in COPD and NS healthy women performed with the Pearson correlation coefficient (r). **(C)** MMP-1; r = 0.4904 and *p <0.0001*. **(D)** MMP-7; r = 0.2902 and *p <0.0013*. Results are expressed as mean ± SD; *p <0.01*. NS (▪). TS (▲). BE (●).

**Figure 2:**
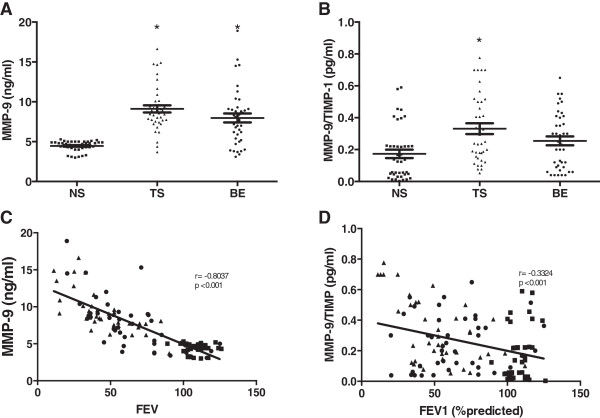
**There were significant differences among BE and TS groups when compared with NS healthy women.** COPD groups did not show any difference: **(A)** MMP-9. **(B)** MM9-9/TIMP-1 complex. Relationship between MMP-9 and MM9-9/TIMP-1 complex with the FEV_1_ (% predicted) in COPD and NS healthy women was performed with the Pearson correlation coefficient (r). **(C)** MMP-9; r = 0.8037 *and p <0.0001;***(D)** MMP-9/TIMP-1 complex; r = 0.2150 *and p <0.0163*. Results are expressed as mean ± SD; *p <0.01*. NS (▪). TS (▲). BE (●).

Plasma concentration of MMP-1, MMP-7 (Figure 1), MMP-9 and the MMP-9/TIMP-1 complex (Figure 2) showed a significant increase both in tobacco smokers and BE groups in comparison with the NS control group; however, no significant differences were found among the COPD groups (Table 2).

**Table 2:** Plasma concentration of MMPs, MMP-9/TIMP-1 complex and C-reactive protein

	**Control**	**BE**	**TS**	***p***
**Molecule**				
MMP-1 (ng/mL)	1.62 ± 0.71	4.24 ± 2.17*	3.86 ± 2.71*	0.01
MMP-7 (ng/mL)	1.79 ± 0.26	2.63 ± 1.12*	2.87 ± 1.26*	0.01
MMP-9 (ng/mL)	4.46 ± 0.65	7.97 ± 3.53*	9.07 ± 2.84*	0.01
MMP-9/TIMP-1 (pg/mL)	0.18 ± 0.17	0.25 ± 0.18	0.32 ± 0.21*	0.05
C-reactive protein (ng/mL)	12.34 ± 6.04	32.89 ± 21.29*	35.49 ± 21.19*	0.05

BE: Biomass exposure; TS: Tobacco smoking.

Data are express as means ± SD. *ANOVA-Tukey test compared with Controls.

### CRP plasma levels in COPD patients

**Figure 3:**
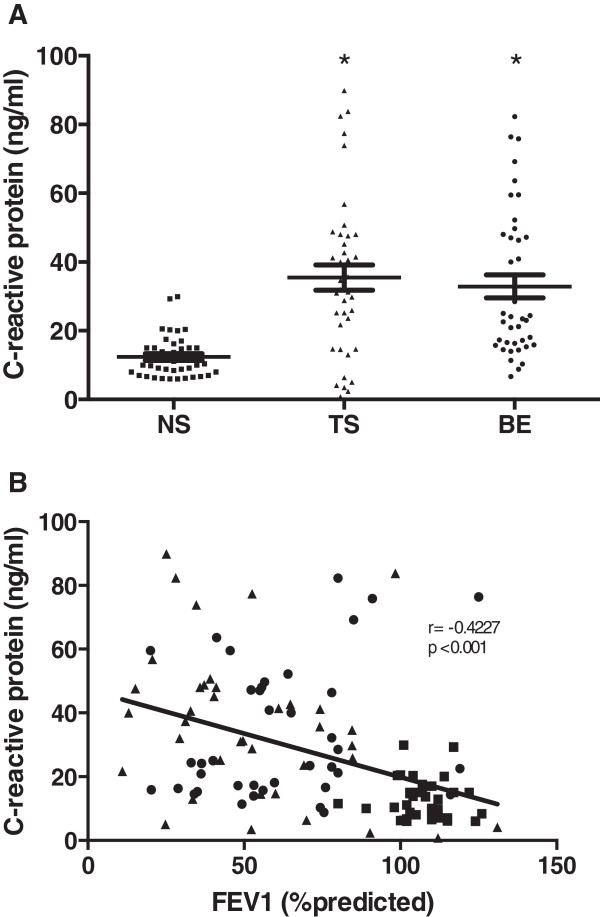
**Plasma levels of the C-reactive protein (CRP) in COPD and NS healthy women, and relationship between CRP and FEV**_**1 **_**(% predicted).** There were significant differences among BE and TS groups when compared with NS healthy women. COPD groups did not show difference among them: **(A)** plasma levels of CRP. **(B)** Relationship between plasma levels of CRP in COPD and NS healthy women with the FEV_1_ (% predicted) performed with the Pearson correlation coefficient (r). r = 0.4227 and *p <0.0001*. Results are expressed as mean ± SD; *p <0.01*. NS (▪). TS (▲). BE (●).

There was a significant increase in CRP plasma levels both in tobacco smokers and BE COPD patients compared with NS control subjects (Figure 3). Despite the scattered picture that can be observed in the obtained data, no significant difference among COPD groups was observed (Table 2).

### FEV_1_ correlations

An inverse correlation between MMP-1, MMP-7, MMP-9, MMP-9/TIMP-1 and CRP plasma concentrations with changes in observed FEV_1_ (% predicted) in both COPD groups when compared to control group was observed (Figures 1, 2 and 3).

## Discussion

The main findings of this work are that: 1) metalloproteinases 1, 7, and 9, the MMP-9/TIPM-1 ratio and CRP concentrations are increased in plasma of subjects with COPD associated to BE, and: 2) an association among these increases and FEV_1_ exists.

Several studies carried out in different populations around the world have established that domestic exposure to biomass solid fuels combustion products is now considered an important risk factor for COPD, mainly in developing countries [3,4,6,18]. A number of similarities including mortality among COPD associated with TS and BE have been observed [3,6]. However, information on the possible role of candidate molecules already involved in the pathogenesis of COPD associated with tobacco exposure is scant in BE [6]. In the present work, we studied the possible role of some of those molecules. All study subjects selected were women because most patients with COPD secondary to domestic BE reported in Mexico are females [4,5,12].

MMPs play an important role in the turnover of almost all extracellular matrix molecules and are therefore probably involved in COPD pathogenesis [8,19]. An increase in serum or plasma MMP-1 and MMP-7 concentration in COPD associated to tobacco smoking has already been demonstrated. Our current findings in BE occur in a similar way, suggesting that these enzymes could be involved in interstitial EM turnover [20-22]. MMP-9 plasma levels and activity have been extensively studied in patients with COPD secondary to tobacco smoke, demonstrating an active role of these enzymes during the inflammatory process that characterizes COPD, especially in the degradation of interstitial and basal membranes molecules of EM, such as types I and IV collagen and elastic fibers [23-25]. Additionally, an increase of MMP-9 has been reported in sputum, blood and lung tissue from smokers with COPD [26-30]. On the other hand, MMP-9 is inhibited by TIMP-1 and an imbalance in the MMP-9/TIMP-1 ratio could be involved in COPD pathogenesis. In this regard, Kang et al demonstrated a correlation among the increase in the MMP-9/TIMP-1 complex in lung tissue from smokers and the airflow obstruction observed [31] and Higashimoto et colleagues [20] found that circulating TIMP-1 concentration was significantly higher in stable COPD patients. This and our report suggest that excess amounts of TIMP-1 compared with those of MMP-9 may be related to airway narrowing. Moreover, our study showed an increase in the MMP-9/TIMP-1 complex that inversely correlated with airflow obstruction in both smokers and BE COPD patients, suggesting a role in COPD associated to BE as it occurs in tobacco smokers.

The increase of CRP plasma concentration has been observed in patients with COPD associated with tobacco exposure as well as in former smokers, suggesting that the inflammatory process persists even when the exposure to a risk factor has ceased [32]. Moreover, Higashimoto et colleagues examined various inflammatory markers where only serum CRP and MMP-9 levels were related to FEV_1_ decline [33]. CRP plasma concentration in biomass smoke-exposed women was previously studied in a group of subjects with COPD related to BE [34]. Although the study sample was small (11 non-smoking subjects), those data and ours suggest that these women develop a systemic inflammation similar to that observed in smokers. In this context, the association showing that the lower the FEV_1_ the higher the CRP levels, supports the possible role of CRP as a biomarker of systemic inflammation in BE. Our results also show that the history and annual rate of exacerbations did not affect the levels of the studied molecules since the frequency is similar in both groups of COPD.

## Conclusions

We report an increase of MMP-1, MMP-7, MMP-9, MMP-9/TIMP-1 ratio and CRP plasma concentrations and a correlation with FEV_1_ in women with COPD associated to BE that is similar to that observed in smokers with COPD. Further research is needed to clarify if these MMPs and the CRP participate in the pathogenesis of COPD in women exposed to BE.

## Abbreviations

BE: Biomass exposure; COPD: Chronic obstructive pulmonary disease; CRP: C-reactive protein; FEV_1_: Forced expiratory volume in the 1st second; EM: Extracellular matrix; FVC: Forced vital capacity; GOLD: Global Initiative for Chronic Obstructive Lung Disease; MMP-1: Matrix metalloproteinase-1; MMP-7: Matrix metalloproteinase-7; MMP-9: Matrix metalloproteinase-9; NS: Non-smoking; TIMP-1: Tissue inhibitor of metalloproteinase-1; TS: Tobacco smoking.

## Competing interests

All authors state no competing interests.

## Authors’ contributions

MM and RS conceived and designed the study conception and design, had full access to all of the data in the study and take responsibility for the integrity of the data and the accuracy of the data analysis. JC contributed in the analysis and interpretation of statistical data, drafting of the manuscript and reading and approving the final manuscript. CB contributed in the analysis and interpretation of biochemical data, drafting of the manuscript and reading and approving the final manuscript. GG-A contributed in the analysis and interpretation of data, drafting of the manuscript for important intellectual content and reading and approving the final manuscript. BS contributed in the analysis and interpretation of data, drafting of the manuscript for important intellectual content and reading and approving the final manuscript. AR-V contributed in the analysis and interpretation of clinical data, drafting of the manuscript and reading and approving the final manuscript. IH: contributed in the analysis and interpretation of biochemical data and reading and approving the final manuscript. LO contributed in the collection of clinical material and reading and approving the final manuscript. CR contributed to study conception and design, analysis and interpretation of data, drafting of the manuscript for important intellectual content and reading and approving the final manuscript. All authors read and approved the final manuscript.

## References

[B1] Tuder RM Petrache I Pathogenesis of chronic obstructive pulmonary disease J Clin Invest 2012 122 2749 2755 22850885 10.1172/JCI60324 PMC3408733

[B2] Goldcopd.org Global Strategy for the Diagnosis Management, and Prevention of Chronic Obstructive Pulmonary Disease Updated January 2014. Available from: http://www.goldcopd.org/guidelines-global-strategy-for-diagnosis-management.html.

[B3] Hu G Zhou Y Tian J Yao W Li J Li B Ran P Risk of COPD from exposure to biomass smoke: a metaanalysis Chest 2010 138 20 31 20139228 10.1378/chest.08-2114

[B4] Pérez-Padilla R Regalado J Vedal S Paré P Chapela R Sansores R Selman M Exposure to biomass smoke and chronic airway disease in Mexican women. A case-control study Am J Respir Crit Care Med 1996 54 701 706 8810608 10.1164/ajrccm.154.3.8810608

[B5] Ramírez-Venegas A Sansores RH Pérez-Padilla R Regalado J Velázquez A Sánchez C Mayar ME Survival of patients with chronic obstructive pulmonary disease due to biomass smoke and tobacco Am J Respir Crit Care Med 2006 173 393 397 16322646 10.1164/rccm.200504-568OC

[B6] Naeher LP Brauer M Lipsett M Zelikoff JT Simpson CD Koenig JQ Smith KR Wood smoke health effects: a review Inhal Toxicol 2007 19 67 106 17127644 10.1080/08958370600985875

[B7] Rylance J Gordon SB Naeher LP Patel A Balmes JR Adetona O Rogalsky DK Martin WJ 2nd Household air pollution: a call for studies into biomarkers of exposure and predictors of respiratory disease Am J Physiol Lung Cell Mol Physiol 2013 304 L571 L578 23457186 10.1152/ajplung.00416.2012 PMC3652022

[B8] Ólafsdóttir IS Janson C Lind L Hulthe J Gunnbjörnsdótti M Sundströ J Serum levels of matrix metalloproteinase-9, tissue inhibitors of metalloproteinase-1 and their ratio are associated with impaired lung function in the elderly: a population-based study Respirology 2010 15 530 535 20337997 10.1111/j.1440-1843.2010.01718.x

[B9] Demedts IK Brusselle GG Bracke KR Vermaelen KY Pauwels RA Matrix metalloproteinases in asthma and COPD Curr Opin Pharmacol 2005 5 257 263 15907912 10.1016/j.coph.2004.12.005

[B10] Shaaban R Kony S Driss F Leynaert B Soussan D Pin I Neukirch F Zureik M Change in C-reactive levels and FEV_1_ decline: a longitudinal population-based study Respir Med 2006 100 2112 2120 16650972 10.1016/j.rmed.2006.03.027

[B11] Dahl M Vestbo J Zacho J Lange P Tybjærg-Hansen A Nordestgaard BG C reactive protein and chronic obstructive pulmonary disease: a Mendelian randomisation approach Thorax 2011 66 197 204 21059738 10.1136/thx.2009.131193

[B12] Menezes AM Victora CG Perez-Padilla R PLATINO Team The Platino project. Methodology of a multicenter prevalence survey of chronic obstructive pulmonary disease in major Latin American cities BMC Med Res Methodol 2004 4 15 15202950 10.1186/1471-2288-4-15 PMC442126

[B13] Rabe KF Hurd S Anzueto A Barnes PJ Buist SA Calverley P Fukuchi Y Jenkins C Rodriguez-Roisin R van Weel C Zielinski J Global strategy for the diagnosis, management, and prevention of chronic obstructive pulmonary disease: GOLD executive summary Am J Respir Crit Care Med 2007 176 532 555 17507545 10.1164/rccm.200703-456SO

[B14] Celli BR MacNee W ATS/ERS task force. Standards for the diagnosis and treatment of patients with COPD: a summary of the ATS/ERS position paper Eur Respir J 2004 23 932 946 15219010 10.1183/09031936.04.00014304

[B15] Pérez-Padilla R Regalado J Vázquez-García JC Reproducibilidad espirométrica y adecuación a valores de referencia internacionales en trabajadores mexicanos demandando incapacidad Salud Publ Mex 2001 43 113 121 11381840

[B16] Hankinson JL Odencrantz JR Fedan KB Spirometric reference values from a sample of the general U.S. population Am J Respir Crit Care Med 1999 159 179 187 9872837 10.1164/ajrccm.159.1.9712108

[B17] Smith PK Krohn RI Hermanson GT Mallia AK Gartner FH Provenzano MD Fujimoto EK Boeke NM Olson BJ Klenk DC Measurement of protein using bicinchoninic acid Anal Biochem 1985 150 76 85 3843705 10.1016/0003-2697(85)90442-7

[B18] Morandi MT Ward TJ The risk assessment workgroup. Biomass risk assessment: defining the questions Inhal Toxicol 2010 22 94 98 20039837 10.3109/08958370903008854

[B19] Chung KF Adcock IM Multifaceted mechanisms in COPD. Inflammation, immunity, and tissue repair and destruction Eur Respir J 2008 31 1334 1356 18515558 10.1183/09031936.00018908

[B20] Higashimoto Y Yamagata Y Iwata T Okada M Ishiguchi T Sato H Masuda M Itoh H Increased serum concentrations of tissue inhibitor of metalloproteinase-1 in COPD patients Eur Respir J 2005 25 885 890 15863647 10.1183/09031936.05.00092804

[B21] Navratilova Z Zatloukal J Kriegova E Kolek V Petrek M Simultaneous up-regulation of matrix metalloproteinases 1, 2, 3, 7, 8, 9 and tissue inhibitors of metalloproteinases 1, 4 in serum of patients with chronic obstructive pulmonary disease Respirology 2012 17 1006 1012 22591289 10.1111/j.1440-1843.2012.02197.x

[B22] Pinto-Plata V Toso J Lee K Park D Bilello J Mullerova H De Souza MM Vessey R Celli B Profiling serum biomarkers in patients with COPD: associations with clinical parameters Thorax 2007 62 595 601 17356059 10.1136/thx.2006.064428 PMC2117244

[B23] Skjot-Arkil H Clausen RE Nguyen QHT Wang Y Zheng Q Martinez FJ Hogaboam CM Han M Kickstein L Larsen MR Nawrocki A Leeming DJ Karsdal MA Measurement of MMP-9 and MMP-12 degraded elastin (ELM) provides unique information on lung tissue degradation BMX Pulm Med 2012 12 34 10.1186/1471-2466-12-34 PMC3515477 22818364

[B24] Simpson JL McDonald VM Baines KJ Oreo KM Wang F Hansbro PM Gibson PG Influence of age, past smoking, and disease severity on TLR2, neutrophilic inflammation, and MMP-9 levels in COPD Mediat Inflamm 2013 2013 462934 10.1155/2013/462934 PMC3628212 23606791

[B25] Noguera A Gomez C Faner R Cosio B Gonzalez-Periz A Claria J Carvajal A Agusti A An investigation of the resolution of inflammation (catabasis) in COPD Respir Res 2012 13 1 9 23148928 10.1186/1465-9921-13-101 PMC3546860

[B26] Ilumets H Rytila P Demedts I Brusselle GG Sovijarvi A Myllarniemi M Sorsa T Kinnnula VL Matrix metalloproteinases -8, -9 and -12 in smokers and patients with Stage 0 COPD Int J Chron Obstruct Pulmon Dis 2007 2 369 379 18229576 PMC2695187

[B27] Ilumets H Mazur W Tojamo T Louhelainen N Nieminen P Kobayashi H Ishikawa N Kinnula VL Ageing and smoking contribute to plasma surfactant proteins and protease imbalance with correlations to airway obstruction BMC Pulm Med 2011 11 19 28 21504569 10.1186/1471-2466-11-19 PMC3103485

[B28] Kang MJ Oh YM Lee JC Kim DG Park MJ Lee MG Hyun IG Han SK Shim YS Jung KS Lung matrix metalloproteinase-9 correlates with cigarette smoking and obstruction of airflow J Korean Med Sci 2003 18 821 827 14676438 10.3346/jkms.2003.18.6.821 PMC3055149

[B29] Brajer B Batura-Gabryel H Mowicka A Kuznar-Kaminska B Szczepanik A Concentration of matrix metalloproteinase-9 in serum of patients with chronic obstructive pulmonary disease and a degree of airway obstruction and disease progression J Physiol Pharmacol 2008 59 Suppl 6 145 152 19218638

[B30] D’Armiento JM Goldklang MP Hardigan AA Geraghty P Roth MD Connett JE Wise RA Sciurba FC Scharf SM Thankachen J Islam M Ghio AJ Foronjy RF Increased Matrix Metalloproteinase (MMPs) Levels Do Not Predict Disease Severity or Progression in Emphysema PLoS One 2013 8 e56352 23441181 10.1371/journal.pone.0056352 PMC3575373

[B31] Kwiatkowska S Noweta K Zieba M Nowak D Bialasiewicz P Enhanced exhalation of matrix metalloproteinase-9 and tissue inhibitor of metalloproteinase- in patients with COPD exacerbations: prospective study Respiration 2012 804 231 241 22832426 10.1159/000339417

[B32] De Torres JP Cordoba-Lanus E López-Aguilar C Muros de Fuentes M Montejo de Garcini A Aguirre-Jaime A Celli BR Casanova C C-reactive protein levels and clinically important predictive outcomes in stable COPD patients Eur Respir J 2006 27 902 907 16455829 10.1183/09031936.06.00109605

[B33] Higashimoto Y Iwata T Okada M Satoh H Fukuda K Tohda Y Serum biomarkers as predictors of lung function decline in chronic obstructive pulmonary disease Respir Med 2009 103 1231 1238 19249197 10.1016/j.rmed.2009.01.021

[B34] Funda A Funda A Nermin Ç Kurtuluş A Ruhsar O Sema C Bünyamin Y Kadir Okhan A C-reactive protein levels are raised in stable Chronic obstructive pulmonary disease patients independent of smoking behavior and biomass exposure J Thorac Dis 2013 5 414 421 23991296 10.3978/j.issn.2072-1439.2013.06.27 PMC3755654

